# Understanding individual and structural factors behind extensive care needs in personality disorders: an interpretative phenomenological analysis

**DOI:** 10.1186/s12991-025-00623-4

**Published:** 2026-02-08

**Authors:** Jennifer Strand, Flavio Di Leone, Evelina Dervisoski, Parinazz Iranmanesh, Peter Sand

**Affiliations:** 1 Department of Psychology, University of Gothenburg, Gothenburg, Sweden; 2 Department of Psychiatry for Affective Disorders, Sahlgrenska University Hospital, Gothenburg, Sweden; 3 Department of Psychiatry and Neurochemistry, Institute of Neuroscience and Physiology, Sahlgrenska Academy, University of Gothenburg, Gothenburg, Sweden

**Keywords:** Personality disorder, Frequent hospitalization, High-care-consuming, Interpretative phenomenological analysis, Qualitative studies

## Abstract

Extensive care needs, including frequent hospitalizations and compulsory care, are common among individuals with personality disorders (PD), yet the outcomes of such interventions remain uncertain. This study aimed to explore patient perspectives on the individual and structural factors contributing to the high demand for psychiatric interventions. Eleven high-care-consuming patients diagnosed with PD were interviewed, and data were analyzed using interpretative phenomenological analysis. The findings indicate that emergency department visits often stem from emotional turmoil and social isolation, reflecting acts of desperation. Participants described inpatient treatments as impersonal, lacking direction, and primarily focused on repeated pharmacological interventions that exacerbated feelings of hopelessness. Both inpatient and outpatient care were perceived as “a state of waiting for something that never happens,” reflecting an ongoing sense of invalidation and unmet needs. These results highlight the urgent need for healthcare systems to prioritize individualized care plans, improved communication, and explore alternative approaches for managing crises or reforming emergency department practices for this group of patients. Addressing these factors could enhance patient well-being, reduce self-destructive tendencies, and alleviate reliance on the mental health system.

**Supplementary Information:**

The online version contains supplementary material available at 10.1186/s12991-025-00623-4.

## Background

Extensive care needs arise when individuals require sustained medical and/or psychiatric interventions to manage severe, persistent, or complex health conditions [1]. In mental health, these needs are often reflected in frequent hospitalizations, prolonged inpatient stays, and recurring episodes of compulsory care. Such patterns of healthcare utilization are typically driven by significant emotional distress, impaired daily functioning, high-risk behaviors, and limited access to personalized or effective interventions [2]. Recurrent and prolonged psychiatric inpatient stays can severely affect patients’ well-being and quality of life, often leading to forms of institutionalization. Moreover, individuals with extensive mental health care needs often face socio-environmental challenges, including unemployment, homelessness, and co-occurring substance dependency.

Extensive mental health care needs have also been linked to elevated risk of suicidal ideation and behavior. Recent evidence indicates that suicide rates have significantly increased in non-European regions, with the highest age-standardized rate observed in the United States (15.5 per 100,000 males), particularly among younger age groups [3].

### Personality disorders and extensive care needs

Although the extent of healthcare utilization varies among individuals, personality disorders (PDs) represent a significant risk factor for high healthcare resource consumption [4]. Among PDs, patients with Borderline Personality Disorder (BPD) stand out due to its significant societal and healthcare costs, which rival those of schizophrenia and exceed those of depression and anxiety disorders [5]. Inpatient care contributes significantly to these costs, and although specialized treatments are available, they often redistribute rather than reduce overall healthcare costs [4]. Interestingly, emergency visits constitute a minor portion of healthcare costs, despite the reputation for frequent emergency service utilization among individuals with BPD [6]. The impact of PDs extends beyond the individuals themselves, demonstrated by increased healthcare costs and productivity losses among spouses of individuals with BPD compared to matched controls [7]. Although BPD is associated with extensive care needs and high healthcare costs, the cost differences with other PD diagnoses are relatively small, suggesting that the broader economic impact is mainly driven by the cumulative burden of untreated or inadequately treated patients across the spectrum of PDs [8].

### Relational dynamics and the “difficult” label

Individuals with PDs who exhibit a pattern of extensive care needs, frequent presentations to emergency and recurrent inpatient care are often regarded as “some of the most difficult cases clinicians encounter in everyday practice” [9]. Every psychiatric clinic has its own stories, equally absurd and dramatic, about “challenging” patients who repeatedly seek help in ways that are perceived as unhelpful, often leaving healthcare providers feeling frustrated and powerless [10].

In the literature, the term “difficult” is used to describe three distinct situations: “difficult-to-treat” patients who typically suffer from psychotic disorders or paranoia, requiring intensive interventions; “difficult people” characterized by antisocial or abusive behaviors that create challenging interpersonal dynamics; and “difficult patients” typically non-psychotic, chronic patients who present significant challenges due to their ambivalent attitudes toward treatment and low compliance with professional expectations [11]. Patients with Axis II disorders, particularly BPD, are frequently categorized within the third group [11]. The ambiguous nature of the "difficult patient" label contributes to diagnostic uncertainty, professional burnout, and potential frequent discharge or referral from care [12].

The term "difficult" is sometimes used synonymously with “severe” in the context of personality disorders, but research has failed to establish a clear association between severity and complexity. For example, treatment for BPD leads to incremental costs compared to the treatment of Major Depressive Disorder, despite the two conditions often presenting with similar severity levels [13]. Moreover, variations in PD severity, measured by the number of co-occurring PDs, the total number of PD criteria, the number of BPD criteria, or the Level of Personality Functioning Scale from the DSM-5 alternative model, account for little of the variation in societal costs within this patient group [5]. This suggests that, despite efforts to identify defining characteristics of "difficult patients," the label fundamentally remains an external judgment, used to describe a specific type of patient-caregiver relationship. “Difficult” patients are frequently associated with traits such as being quarrelsome, intransigent, and provocative [14]. These individuals are frequently held responsible for evoking strong countertransference reactions in their care providers, leading to treatment deadlocks and the use of unnecessary interventions [15]. Such dynamics can escalate into power struggles over medication, privileges, or admission decisions, ultimately disrupting the therapeutic relationship and halting progress [16].

It can be theorized that extensive care needs, which often manifest as a pattern of recurrent, unsuccessful attempts to seek help, particularly among individuals with PDs, points to underlying disturbance frequently summarized under the label “difficult.” In this context, the term can encompass (1) the difficulty in reaching out to the patient, (2) the challenge in managing recurrent or disruptive symptoms, and (3) the ambivalence patients seem to experience in seeking help [16].

If the “difficult” label correlates with the extended care needs of patients with PD but does not necessarily reflect the severity of their condition, it raises important questions about the factors that contribute to this perception. Additionally, it is important to examine how the healthcare system responds to these needs and whether these responses contribute to or reinforce the behaviors that lead to patients being labeled as “difficult.”

### Study aim

The present study aims to explore how individuals with personality disorders and extensive care needs experience and make sense of their repeated encounters with psychiatric care. Specifically, it seeks to understand how personal meanings and structural conditions interact in shaping the continued demand for psychiatric interventions.

## Method

This study used a qualitative design with semi-structured in-depth interviews to explore the experiences of patients with PD who frequently sought psychiatric inpatient care or visited the psychiatric emergency department.

### Clinical context

The study was carried out at the Department of Affective Psychiatry, Sahlgrenska University Hospital in Gothenburg, Sweden. Participants were recruited from four different outpatient services that cater to a metropolitan population of 770,000 residents and specialize in mood disorders, anxiety-related issues, post-traumatic stress disorders, eating disorders, and personality disorders.

At the time of recruitment, the Department of Affective Psychiatry had four specialized outpatient services, each with dedicated teams for affective disorders (such as anxiety, depression, and PTSD), neuropsychiatric disorders (including ASD and ADHD), and personality disorders (PD). Teams included 6 to 16 healthcare professionals, including 1 to 4 psychiatrists.

PD patients were usually treated by the specialized PD team, but alternative pathways were possible based on treatment history and comorbidities. PD teams conducted assessments and provided various psychological interventions: Mentalization-Based Therapy (MBT) and Dialectical Behavioral Therapy (DBT). Psychodynamic and cognitive behavioral therapies were offered individually or in adapted forms for those not suited to MBT/DBT.

Outpatient services served were the primary in emergencies, although a 24/7 psychiatric emergency department handled acute crises, hospitalizations, and compulsory care. Emergency staff did not prescribe medications or provide ongoing support, referring patients to their outpatient contacts. Inpatient care was provided across four general psychiatric wards.

### Participants

To identify individuals with extensive care needs, the Department established three arbitrary yearly thresholds: (1) five or more visits to the emergency department, (2) three or more hospitalizations, and/or (3) more than 60 cumulative days of inpatient care. Individuals who met these criteria and had at least one diagnosis of PD were considered eligible for the study. The following exclusion criteria were applied: Patients with severe psychiatric comorbidities, such as bipolar disorder, substance dependence, psychosis, and acute suicidality at the time of recruitment, were excluded, as were patients involved in an ongoing custody dispute.

Between 2019 and 2022, individuals with PD and extensive care needs were observed in 8–11% patients. Fifty-five were eligible to participate in the study and 11 accepted. We have no information about those who did not accept to participate. Clinical and demographic data are presented in Table 1.

**Table 1:** Demographic and clinical data of participants

Participant ID	Gender	Age	Diagnosis
1	Female	20	EUPD
2	Female	25	EUPD
3	Female	30	EUPD
4	Female	35	EUPD
5	Female	40	EUPD
6	Female	45	EUPD
7	Female	50	PD-UNS
8	Female	52	PD-UNS
9	Female	38	DPD
10	Male	28	INCD
11	Male	32	INCD

Gender, age, and primary diagnosis of the 11 participants

PD-UNS = Unspecified Personality Disorder

EUPD = Emotionally Unstable Personality Disorder

DPD = Dependent Personality Disorder

INCD = Impulse Control Disorder

### Data collection

The participants were contacted either by the primary investigator (PS) or by the interviewers. During this initial contact, a time was set for the data collection. Participants also received verbal information about the study’s aim and ethical guidelines. At the time of the interview, the participants received written information about the study and signed an informed consent. All interviews were scheduled and conducted at one of the clinic’s outpatient units.

A semi-structured guide was used during the interviews (see Appendix for interview guide). All questions aimed to capture aspects of the overall aim, e.g., the patient’s perspective on how individual and structural factors lead to extensive care requirements among individuals with PD. Examples of interview questions are: Is there anything within yourself that has contributed to the high frequency of inpatient care? Is there anything related to your care that have contributed to the high frequency of inpatient care? Are there any specific interventions that could prevent you from needing inpatient care? Follow-up questions were used to deepen and clarify the informants’ answers.

All interviews were audio recorded. Seven were conducted by one licensed psychologist (PI) and four by one psychologist (ED) undergoing clinical training in psychology at the psychiatric clinic’s emergency department. They received similar instructions, but there were some differences in their interview style. The first interviewer asked more follow-up questions, which lead to more reflective and detailed answers, making those interviews somewhat more extensive. Apart from these differences, there were individual variations in how talkative the informants were, and the length of the transcribed interviews varied between four and 17 pages.

### Analysis

The data was analyzed using Interpretative Phenomenological Analysis (IPA), following the guidelines by Smith et al. [17]. The underpinnings of IPA—phenomenology, ideography, and hermeneutics—were regarded as suitable and adhered to at different stages of the analysis.

The phenomenological approach is embedded in both the research question and the interview questions, reflecting our intention to capture the patients’ subjective experiences and their sense-making processes regarding their extensive use of inpatient care. The phenomenological focus extended to the first round of the coding and significant statements that reflected how the participants experienced factors that contributed to their demand for inpatient care were noted. The codes were close to the transcripts in wording and meaning as the intention was to capture the essence of participants’ experiences as authentically as possible. The phenomenological coding was done by one of the researchers (JS).

The ideographic approach in IPA was acknowledged by the case-by-case analysis. Each transcript was coded with unique codes and a short summary for each interview was written. Every participant’s account was treated as unique, and their personal meaning-making was attended before searching for patterns across cases. This ideographic approach also influenced the write-up of the results, ensuring that individual experiences were presented in their unique context.

The hermeneutic turn was used to examine relationships between individual parts (e.g., codes and themes) and the broader narrative (e.g., overall themes). This involved exploring connections within each interview and between interviews. Our intention throughout the analysis and write up of the study was to balance between how the informant make sense of their experiences and how we as researchers make sense of their experiences. The themes and the relationship between them were discussed in the research group.

The analysis continued throughout the writing of the results section, and differing interpretations of participants’ experiences were resolved through collaborative discussion and by testing alternative readings against the data. During the writing process, two subthemes were merged into a single theme, as repeated patterns in the data revealed significant overlap between them. This procedure resulted in four main themes with two to three subthemes respectively.

### Ethics and reflexivity

The study was approved by the Swedish Ethics Review Authority (reference nr: 2020–07105). We followed formal ethical guidelines, but other ethical situations arose. For example, many informants were upset by how they had been treated in psychiatric care; others cried and expressed hopelessness and despair. Some seemed to approach the interview as an opportunity to express dissatisfaction with their care. One informant participated in the interview because she hoped it would improve her care, and two perceived the interview as an opportunity to make complaints about specific psychiatrists and treatments. It is possible, that some of these situations could have been avoided if the difference between research and health care had initially been clarified in more detail.

Our experiences and preconceptions of psychiatry and psychiatric care varied within the research group, as we come from different scientific traditions such as medicine and psychology. These differences gave rise to discussions about these patients care needs and how treatment should be organized. Hopefully, our different perspectives have enriched the data and contributed to a nuanced interpretation of the interviews.

## Results

The results are structured into four main themes: 1. The tipping point that leads to…, 2. …an encounter without subjects, 3. A long walk… 4. …without ending. Each main theme is structured into subthemes (insert Table 2 here), see Table 2. The intention of the thematic structure is to capture the essence in each theme and present them in a timeline that reflects the starting point of the informants’ need for hospitalization, through their encounters with inpatient and outpatient care, and finally to their current situation as patients in psychiatry. To preserve anonymity, all informants have been given pseudonyms.

**Table 2:** Main theme and subthemes

Main theme	Subtheme	Subtheme	Subtheme
1. The tipping point that leads to…	1.1 Emotional turmoil	1.2 Fear of self-harm and suicide	
2. …an encounter without subjects	2.1 The last resort	2.2 A struggle to get pass	2.3 To stay or to leave
3. A long walk…	3.1 Failed communication	3.2 Treatment without target	
4. …without ending	4.1 Hopelessness	4.2 Lack of agency	4.3 Trapped

## The tipping point that leads to

This theme encompasses experiences of events and emotions that led to hospitalization. Relational collapses could cause rapid and uncontrollable emotions, eventually leading to self-destructiveness and suicidal thoughts or attempts. For all informants, seeking psychiatric inpatient care was an act of desperation in a relational vacuum.

### Emotional turmoil

The importance and risk of interpersonal relations were evident throughout the interviews, reflected in the similarities of stories about what had preceded the emotional breakdowns that led the informants seek inpatient care. The emotional turbulence triggered by relational issues was exacerbated by the lack of emotional support they longed for. All but one informant described how relational collapses, most commonly with a partner, caused a loss of emotional control: “Then she broke up, and everything fell apart. I didn’t have anything ”. (Anders)

The period that preceded psychiatric inpatient care resembled a reinforcing vicious circle. Camilla and Fiona described that escalating anxiety led to social withdrawal and isolation that strengthened hopelessness and caused increased anxiety and eventually panic attacks: “I want to get out and when I get out, I just want to disappear. I have a hard time controlling myself, others have control, but I don’t. I can’t handle it; I get panic and anxiety”. (Fiona).

During turbulent periods, the informants seemed trapped in a chaos of emotions they were unable to cope with. Parallel to descriptions of anxiety, they also described feelings being unreal and they questioned the validity of their emotions.

That circle is quite self-sustaining, it breeds itself. Yes, but for me it’s often that it starts with me feeling that I’m not... something that can become quite big is that I feel like I’m not ill enough or that I don’t get validation and then it helps with self-harm. (Camille)

### Fear of self-harm and suicide

Self-harm and suicidal ideations often came from overwhelming emotions experienced in the absence of supportive relations. The fear of eventually crossing the line and commit suicide pushed some to seek help in emergency care.

Eva, as many other informants, described fear of self-harm and suicide but had no other ways of restraining herself than turning to the emergency department. She was persistent in her attempts to be hospitalized and described herself as stubborn: “I try to stay away from inpatient care because I’m afraid of it, but at the same time I’m stubborn and I don’t want to go as far as to actually hurt myself”. Anders described his will to survive as a “parasite” which made him turn to inpatient care rather than completing his suicide plans.

Others described the suicide plans as an obsession they felt unable to control. For Camilla, it was the fear of uncontrollable thoughts that made her search inpatient care: “There is some kind of fixation on suicide and stuff like that, it becomes all I can think of. It’s like I kind of long and hope and push myself to it.”.

## An encounter without subjects

This theme explores the informants’ experiences of encountering inpatient care, and the subthemes are organized chronologically. The first theme includes their feelings when finally turning to inpatient care, their perceptions of how they were approached at the emergency department, and the third their experiences in the psychiatric ward. The subthemes are characterized by the perception that the interaction between the patient and the professional, especially the psychiatrist, were a meeting without subjects. The informants felt reduced to “a patient”, the psychiatrist was perceived as a gatekeeper, and the psychiatric services were described as “cold”, “stereotyped”, and “inhuman”.

### The last resort

Inpatient care represented the last resort; a place they turned to when all other possibilities were exhausted. Seeking inpatient care was experienced as the final failure: “I don’t want to be hospitalized, I try as much as I can at home, to use the skills I have” (Kim). Although the stories shared similarities, Dana’s narrative stood out in desperation. Despite ambivalent feelings against inpatient care due to an incident at the ward, she continued to turn to inpatient care in lack of other alternatives.

And I don’t really dare seek treatment, but I have no other options. I’m afraid more things will happen there in the hospital, I don’t trust the doctor if something happens there. The fact that I’m in the room and a guy grabs my breasts might happen again and I’ll get more trauma. (Dana)

Except from describing inpatient care as the final failure, some perceived it as lifesaving and the last option after all other attempts to get help had failed.

I called these suicide lines and everything, so the only alternative was to be hospitalized, and then I sort of got away from those plans I had at home. Because at home, everything became a weapon; a bathtub became a weapon. Everything—pills, alcohol, knives—became a weapon." (Anders)

### A struggle to get past

All informants shared frustrations about the challenges they faced when trying to gain admission to the ward. Not being admitted was described as a “rejection” by the healthcare system rather than an assessment of their needs. The procedures did not align with their mental health needs, and the admission procedure was perceived as unpredictable.

It depends on who you meet, whether you are admitted or not; it does not depend on how I feel. I could go there with a knife and cut myself while I’m talking to them. Then I’m a little clearer maybe, but ideally you want to do it with just words. (Ben)

Informants felt uncertain about the motivation behind the decisions. The majority asserted that when seeking emergency psychiatric help repeatedly, their actual needs were “neglected”, and the response was contingent on their previous status, diagnosis, or outcome to previous hospitalization: “When you come to inpatient care, you are not treated as a human being, you are a borderline. I don’t think you can go by a diagnosis and treat everyone the same” (Camilla). Such preconceptions, rather than their mental health statues, became their understanding of reasons for not being admitted to the ward.

The experience of not being hospitalized became a “rejection”, validating the unpredictably and unreliability of the system. Informants used words such as “painful”, “devastating”, “not fun”, “humiliating” and “escalating hopelessness” to describe this experience. The “rejection” seemed to worsen their self-destructiveness, and Dana described a situation in which she had decided to follow through with her plan after not receiving the help she wanted:

Once in the emergency, I said that I feel so bad that I need to talk to someone. They said there isn’t anyone, so I went to a bridge to jump. Then a guy came, and he called the police and then they took me down. I don’t want to end up in such situations, to take my life that way.

### To stay and to leave

When admitted to the ward, another struggle began. The psychiatric ward was at the same time desired and feared and informants described ambivalence to whether they should stay or leave.

For some, this ambivalence began when they felt the need for inpatient care and thought about the risks it entailed: “There’s sort of a consensus, going to the psych ward, there’s about a 30% chance that something will get better and a 70% chance that something will get worse. And once I’m there, I just want to leave” (Eva).

Maria, reflecting on the end of a supportive therapy at the ward, expressed her conflicting feelings: “It was my only safe connection in life, but then the problem was that when the hospitalization ended, because it took a long time to trust her, I didn’t want to go back. […] It’s worse when they treat you badly because you have a closer relationship.”

The ambivalence towards the ward had different explanations. Some described the environment as too chaotic, overcrowded and understaffed. Others explained that their urge to leave increased due to loneliness and a lack of social support. Gina added that the impersonal, dull atmosphere made her feel worse, intensifying her desire to be discharged. For others, it was the combination of unresponsiveness and dissatisfaction with care that made them sign out from the ward, and in some cases, return.

It happened twice that I signed out and I had to go back. And then I felt it was a mistake, came back and got better care and maybe was more receptive. Maybe that’s why it was so many times too. Partly, it was because of myself, but also my experience there which was not so good. (Camilla)

## A long walk

This theme encompasses experiences of the emergency ward, inpatient and outpatient care, and relates to the informants’ perceptions of how they were approached by the staff. The essence in the first sub-theme includes experiences of feeling unheard and invalidated, leading to a sense of mistrust. The second subtheme concerns their treatment and involves narratives about care perceived as unstructured.

### Failed communication

This is the largest theme in terms of the number of quotations and underscores the pervasive challenges in the communication between patients with personality disorders and psychiatric care settings.

When the informants gave examples of failed communication or experiences of not being listened to, it mostly involved interactions with psychiatrists. Ben’s story is one example of many that reflects the consequences of failed communication. He had been on and off medications for many years. However, during meetings with doctors, he experienced standardized treatment procedures despite his attempts to communicate his personal experiences.

Then the long walk began, feeling like a fucking lab rat, testing all kinds of meds although I had told them what had worked for me. So, I had to try medicine, I was probably up to 12−10 medicines everyday and... I was completely crazy, resulting in more visits and hospitalizations. (Ben)

Fiona’s perception reflects experiences described by all informants; establishing mutual communication and interpersonal relations was difficult: “When I meet a doctor, it feels like sitting with a table, no feelings. Just tapping on the computer and then; “Goodbye”. Camilla highlights the common perception among patients that medical records and documentation often hold more sway than their own lived experiences and self-reported symptoms.

It feels like they trust the journal more than the patient. If one doctor has misunderstood something the next doctor trusts the journal more than they trust me. And I feel that doctors do not listen very well, they make their own interpretations and decide it’s the right one. It becomes very difficult to struggle against a journal that does not reflect how it is (Camilla).

Mistrust emerged as a direct consequence of communication failures. Informants felt their concerns were disregarded in favor of predefined diagnoses, with all their behaviors and feelings automatically interpreted as symptoms. Such interpretations created feelings of not being seen as a person, which in turn led to a lack of trust in decisions.

### Treatment without target

Two aspects stood out in this theme: waiting and dissatisfaction with changes in prescribed medications that were not recognized as helpful. The informants waited for various interventions such as appointments with psychiatrists, changes of psychiatrist, unit transfers, alterations in medication, and to see or change psychologist. Some had waited for years, and it seemed as though they waited for something that never materialized: “I waited 12 years for therapy, but it never happened/…/it’s just a long wait and no one knows the way forward” (Jenny). For others, it seemed difficult to accept that there might not be a “cure” for their suffering.

Some informants expressed bitterness towards following medical advice and in their view, wasting time on interventions they not only perceived as unhelpful, but also damaging.

Because there is an enormous bitterness these days, like 20 years of my life, I’ve been on medicine, and it hasn’t given me anything but side effects and (laughter). I should have realized that long before. But I have realized that after 20 years and I will never go back, ever. (Eve)

Others described losing direction in a system with different interventions, each profession convinced that their method was the key to recovery. Anders, despite knowing what he needed, felt directionless during periods of anxiety and depression in a care system that he described as a “complete mess”.

I would like to write a book on the tips I’ve been given about everything I should do, everyone has their religion which they push on you, some think it is very important with diet and food and some that you should get out and my physiotherapist thinks that "as long as you exercise properly, you don’t need to take medicine" and the doctors think "as long as you take your medicine properly you don’t need to...". (Anders)

Hanna experienced that her treatment lacked direction due to the aimless conversations she had with her care provider: “It feels like we are having a cup of coffee and don’t talk about serious stuff, and time just passes. I want us to talk for real and go deeper into how I feel and how to deal with my anxiety”.

The disconnection between subjective experiences and clinical realities seemed to create alienation, with individuals feeling estranged from their own care: “I’ve always felt that I’m too complicated for the healthcare system because I don’t really fit the mold and the diagnoses “. (Ben).

## Without ending

This final theme synthesizes the key findings from the preceding main themes and intends to capture the consequences of the encounter between the informants’ problems and needs, and the care system. The first sub-theme encapsulates narratives of hopelessness, the second conveys lack of agency, and the third reflects the perception of being trapped within the care system.

### Hopelessness

All narratives encompassed hopelessness. The informants appeared to oscillate between moments of hope for improvement, periods of hopelessness and suicidal ideations. However, after many years within psychiatric care and still not feeling improved, their stories were mostly filled with resignation and despair.

I would have liked more variation and more like... I would have felt more hope if I knew that there was more to do. Because I have, or it has hardly felt like there has been any plan, so I haven’t understood how to be able to have a normal life even, or if there is a plan for it. (Camilla)

Also, Anders’ narrative reflected hopelessness. He experienced episodes of depression and suicidal ideations and as an alternative to suicide, he had decided to try electroconvulsive treatment. This intervention not only proved ineffective but also resulted in adverse side effects and memory impairment.

I’ve always taken a lot of medication and it never helped, so they said that it might be worth a try. I’m terrified of memory loss but they said "Well it won’t happen", and that’s exactly what happened (laugh). I also woke up during a treatment, it wasn’t funny, because you get muscle relaxants and stuff like that so I couldn’t communicate, so I kind of lay awake and got this electric treatment. But now I can draw a line for it, now I have tested, and I will never do it again. However, another door is closed now, that emergency solution no longer exists. And then the question is: what can I do next time? When I end up there [in depression] because I will end up there again. (Anders)

Besides expressing hopelessness, Eva and Ines narratives were marked with anger against psychiatry and bureaucratic processes. However, Eva expressed that taking the fight required an energy she did not have: “It feels like we’re fighting a war we can’t win”.

### Lack of agency

This theme includes narratives that, to various degrees, could be interpreted as a lack of agency and tendencies to attribute responsibility to external factors. These tendencies varied, and there were individual differences in the extent to which responsibility for actions and lack of progress were caused by psychiatric care. Ines, for example, attributed her lack of progress to failures in care, placing the responsibility outside herself.

I got DBT for my borderline, but then I developed symptoms more like PTSD, and DBT hasn’t helped with that, so either way it’s a failure of care if I don’t get better. Not by me per se, but there’s something in the care that has not been enough in any case. (Ines)

For others, psychiatric care was perceived as liable for their lives and the responsibility for one’s own actions seemed externalized. Kim attributed responsibility for her actions to the doctor who prescribed her medications: “They let me out [of inpatient-care] with a bag of medication and didn´t tell my assistants, anything can happen with those tablets”.

Another example interpreted as externalization of one’s actions is Fiona’s description of an event at the ward. She explained a situation where she jumped from a balcony and then underlined that this could have been avoided if the door had been locked: “I jumped from their balcony while I was under compulsory care with continuously observation. The door was open, so I jumped, and I shouldn’t have been able to do that, really”.

Some informants associated their lack of agency with periods of depression and anxiety, during which they felt disoriented both internally and due to difficulties in communicating their needs*.* Others articulated their internal struggle to assert themselves as active participants in their own lives as "stubbornness," "perfectionism," and "fairness".

The sentiment above highlights a struggle with hopelessness and a strong inclination towards problem-solving and resolution. Camilla shared a similar sentiment, revealing,

I have a hard time letting things go. I think it’s because I’m used to getting what I want... and I want to solve things, but I was used to getting what I wanted in many areas, and when it doesn’t really work out, it goes wrong. I have a hard time letting things go and accepting things. I don’t really know.

### Trapped

This final theme highlights the informants’ sense of entrapment in the healthcare system, as they longed for meaningful progress and support that remained out of reach. They perceived themselves as lacking the capacity and conditions for improvement, leaving them reliant on a system that was too inattentive and overburdened to address their individual needs effectively. For many, this combination seemed to pacify them and leave them in a state of limbo. They felt stuck in a cycle of care that they desperately sought but, paradoxically, found more harmful than helpful.

It just feels like the care is quite narrow, there’s only one way, and if that doesn’t work, there’s hardly anything else. I see a psychologist once a week, and then if things get difficult, it’s the psych ward. I would have liked more variety and more... I would have felt more hopeful if I knew that there was more to do. (Ines)

Lacking other alternatives, many continued with the treatment they were offered, while some struggled with different medications and moved back and forth between inpatient care and hospitalizations: “But then you get stuck in the shit and take medications against medications and then there is no way out but because you don’t dare to stop with the crap even though you actually know it won’t help. (Ben).

Others described a heightened fear associated with inpatient care, particularly of restraints. However, lacking options to manage anxiety, suicidal ideations, and self-harm, they continued to return, as no viable alternatives were available.

And then I get belted maybe 10–15 times, I don’t really remember. I find it annoying that that bed comes out in front of my face. Sometimes they have kept me belted unconscious for 4 hours/…/but I want them to do a proper examination and say "we can make you well or we can’t". I get no answers for years. Nothing works and I’m kind of stuck in something that makes me worse. (Dana)

Another facet of feeling "trapped" involves some informants’ fear of progress. Eva, for instance, worried that improving her situation might lead to a loss of the support she currently relies on. This fear of losing their safety net seems to hinder some informants potential improvement.

Finally, Kim’s quotation will end this theme as it reflects the essence in many of the narratives that forms its foundation: “You’re trapped in a state waiting for something that never happens”.

## Discussion

The aim of this study was to explore the individual and structural factors that contribute to the extensive need for care among individuals with personality disorders. The results will be discussed in relation to previous research, with attention to their clinical relevance and suggestions for future studies. However, before presenting the findings and drawing conclusions, we wish to emphasize the limited number of participants and the possibility that those who chose to participate may have been more dissatisfied with the care they received.

### Interpersonal triggers and emotional dysregulation

The first theme, "The tipping point that leads to…" and "…an encounter without subjects," reflects the phenomenology of interacting with the health care during times of crisis. Informants described difficulties with self-regulation, often escalating into self-destructive behaviour or suicidal ideation. For all, seeking psychiatric inpatient care had been an act of desperation, carried out in a relational vacuum. Emotional turmoil is well-documented in BPD [18] and may explain the recurrent crises reported here. Clinically, these findings underscore the need for brief, relationally attuned crisis interventions that validate distress while discouraging repeated crisis-driven presentations. Staff training in validation, empathy and containment could reduce escalation and strengthen trust between patients and caregivers.

### Experiences of emergency and inpatient care

In line with previous research [19], our findings indicate that emergency care were sought due to the need for human contact, loneliness, or unmet support rather than acute medical risk. Social isolation and unemployment, well-established predictors of emergency attendance [20], were frequently present. There were also recurrent descriptions of shame and withdrawal linked to unfulfilled life goals, with loneliness emerging as a key trigger for crises.

Encounters with emergency and inpatient care were commonly perceived as invalidating or dismissive, echoing previous reports of stigmatization and inconsistent treatment [20]; [21]). Participants experienced the emergency department as a gatekeeping arena, where they were required to “prove” their worth to access care. This was particularly challenging for individuals with an unstable sense of self, as highlighted by Brännström et al. [22], since the demand to articulate and justify one’s need for help often collided with feelings of inner fragmentation and self-doubt. Open and validating communication, transparent triage procedures, and coordinated transitions to outpatient care could improve patients’ understanding of why emergency care was denied. Such clarity may reduce the risk of escalating self-destructiveness and repeated emergency visits triggered by experienced rejection.

Consistent with previous research on stigmatizing attitudes and guideline ambiguity [16]; [23]; [24]), our findings show that judgmental or inconsistent professional responses perpetuate mistrust and exclusion. Such inconsistency points to a lack of shared standards and reflective support for staff, highlighting why structured protocols and regular supervision are needed to ensure relational competence, de-escalation, and stigma reduction. Similarly to Sand et al. [25], experiences of coercive or symptom-worsening medication decisions further illustrate the importance of transparent, collaboratively negotiated treatment goals.

### System entrapment and structural barriers

The themes of “A long walk…” and “…without ending” shed light on the frustration of navigating the healthcare system. The themes reveal how perceived unstructured care without clear directions create feelings of being “stuck” in care, and risks fostering hopelessness, dehumanization, and suicidality [26]. Participants emphasized the importance of receiving clear information about their assessment, diagnosis, and treatment plans, an element repeatedly shown to enhance engagement [27]. However, informants described persistent communication failures and mistrust of psychiatric institutions, a challenge consistent with the literature on barriers faced by individuals with PD [28]).

For many, emergency care served as a gateway to further help yet often became a revolving door due to weak or fragmented outpatient follow-up. Additionally, limited efficacy of evidence-based psychotherapies for PD [29] may reinforce hopelessness and disengagement. Clinically, stronger continuity mechanisms such as coordinated transfer of care, designated case managers, and scheduled post-crisis contacts could reduce reliance on emergency services. To counter this, stronger continuity mechanisms such as designated case managers, scheduled post-crisis contacts, and integration of social and occupational interventions are needed. Emergency psychiatric care could also meet support needs arising from social isolation, offering temporary relief in the absence of stable relationships or community ties. Possibly, programs like supported employment, housing support, and community-based care could address social isolation, enhance agency, and reduce reliance on emergency services as shown by Beeney et al. [30] and Liljedahl et al. [31].

Participants’ descriptions of being refused care or labelled as “difficult” reflect a form of structural exclusion, where patients are formally recognised as in need but practically denied access to appropriate support. This dynamic corresponds to Sulzer’s [32] concept of *demedicalisation*, in which the diagnosis of BPD is acknowledged within psychiatry yet effective treatment pathways remain obstructed. Reducing exclusion requires transparent admission criteria and alternatives such as self-admission models that enhance autonomy without reinforcing dependency, as has been suggested in previous studies (Tan & Hope, 2025; [33]. However, as Newton-Howes [34] and Lundahl, Helgesson and Juth [35] point out, balancing this autonomy with the ethical and practical need for temporary containment remains a persistent dilemma in psychiatric care.

Taken together, findings demonstrate how individual vulnerabilities, emotional dysregulation, shame, and loneliness interact with structural shortcomings, stigmatization, fragmented care pathways, and limited continuity to sustain a cycle of crisis and readmission. This intersection highlights leverage points for service improvement and staff training. From an interpretative perspective, informants’ narratives reveal a paradox of care: systems designed to provide safety and stabilization may, through rigidity, lack of clear treatment goals, and stigma, perpetuate dependence and crisis-driven help-seeking. At a structural level, these insights point to gaps in current service provision and areas where evidence remains insufficient. Evidence for crisis interventions specifically tailored to adults with BPD remains limited, highlighting a persistent research gap identified by Monk-Cunliffe et al. [36]**.** Combined with resource constraints, this lack of evidence continues to challenge emergency care practice. Despite these limitations, prior studies have shown that training in validation and empathy improves communication skills and strengthens therapeutic alliances [37], while collaborative treatment planning helps align care with patient goals [38]. Furthermore, Sand [25] observed that medication decisions in this population are often motivated by the wish to build rapport rather than by therapeutic efficacy, underlining the need for clear prescribing guidelines. Finally, developing comprehensive protocols for managing PD and comorbidities has been suggested in previous research to reduce overmedication, enhance provider trust, and empower patients through transparent planning, thereby reducing stigma and improving outcomes [23]; [21]).

These interacting factors are summarised in Table 3.

**Table 3:** Factors contributing to extensive care needs

Factor type	Description
Individual factors	Social isolation and lack of meaningful societal roles Ambivalence and mistrust regarding treatments, providers, and the support system Struggles to gain or delegate responsibility and agency Difficulties with emotional self-regulation, such as managing intense emotions and impulsive reactions
Systemic factors	Failed communication between patients and providers, especially doctors Unclear procedures, policies, and treatment plans leading to patient dissatisfaction and mistrust

The table outlines the individual and systemic factors contributing to the extensive care needs of participants with personality disorders

In conclusion, the findings highlight how intertwined individual vulnerabilities and structural conditions shape care dependence. This understanding provides a context for reflecting on the methodological approach and its limitations, discussed below.

### Methodological limitations

The study has methodological limitations that require consideration. The coding was primarily conducted by one researcher, which aligns with the idiographic and interpretative nature of the approach. However, it can also raise questions about subjectivity. The researcher intended to maintain a reflexive stance throughout coding, and discussions with colleagues were used to critically reflect on emerging themes and interpretations. Furthermore, we constantly strived to bracket our preconceptions about personality disorders while also taking advantage of our knowledge. Using a combination of a phenomenological approach, focusing on the informants’ subjective perspectives, and the hermeneutic interpretation facilitated this balance.

The relatively small sample size aligns with IPA’s idiographic focus, which emphasizes in-depth exploration of individual experiences rather than generalization. However, the length of the interviews varied between four and 17 pages. This variation reflects not only differences between the interviewers, but also the informants’ varying willingness and ability to provide detailed and in-depth descriptions of their experiences. Furthermore, the sample primarily consisted of individuals with emotionally unstable personality disorder, which may have influenced the thematic emphasis and limited the range of perspectives captured. Possibly, recruiting more informants could have resulted in different, perhaps more positive, experiences of psychiatric care. It is also likely that patients who agree to participate in a study are more dissatisfied with care and perceive the interview as an opportunity to express their complaints.

## Conclusion

In summary, this study contributes to a deeper understanding of the complex interplay between individual vulnerabilities and structural barriers that shape the extensive care needs of individuals with personality disorders. By centering the perspectives of individuals with lived experience, our findings offer insights into both the personal struggles and systemic shortcomings that drive high psychiatric service utilization.

Our findings illustrate how relational triggers and emotional dysregulation frequently escalate into crises, driving individuals toward emergency care, often in the absence of sufficient outpatient support. At the same time, the structural limitations of psychiatric services, including stigmatization, unclear treatment pathways, and limited access to long-term interventions, further entrench reliance on emergency care.

Breaking this cycle requires a paradigm shift in how care is structured and delivered. Interventions should extend beyond symptom management to address social isolation, promote meaningful engagement in society, and enhance patients’ sense of agency. Strategies such as self-admission models, supported employment programs, and community-based care have the potential to provide more sustainable support while reducing emergency visits. Additionally, improving communication between patients and healthcare providers is crucial in fostering trust and engagement in long-term treatment.

### Future directions for research

This study underscores the need for a more patient-centered and structurally responsive approach to psychiatric care. Future research should focus on evaluating the effectiveness of alternative care models, addressing gaps in crisis interventions, and developing strategies to ensure individuals with personality disorders receive timely, equitable, and comprehensive care.

A clearer definition of "extensive care needs" among individuals with BPD should be prioritized to better understand the factors that differentiate high utilizers from average utilizers. Identifying these distinctions could provide valuable insights into patterns of service use and inform more effective allocation of resources.

Another critical area of exploration is the differentiation between non-responders to treatment and individuals who have not yet received adequate care. Investigating the unique experiences and needs of these groups could help refine treatment approaches and ensure interventions are better tailored to individual circumstances.

Additionally, research should evaluate the role of emergency rooms in managing mental health crises for this population. Exploring alternative functions, such as providing "psychological first aid" or brief counseling services, could offer a more therapeutic and supportive approach during crises, potentially reducing dependence on emergency services and improving immediate care outcomes.

## Supplementary Information

Additional file 1.

## Data Availability

The data analyzed in this study consist of participant quotations embedded within the text. Additional data are not available due to confidentiality considerations.
