# Supplementary material for: Understanding individual and structural factors behind extensive care needs in personality disorders: an interpretative phenomenological analysis
Source: Ann Gen Psychiatry. 2026 Feb 8;25:15. doi: 10.1186/s12991-025-00623-4 (PMC12930921; doi:10.1186/s12991-025-00623-4)
Supplement: Supplementary file 1 — Additional file 1. [file 12991_2025_623_MOESM1_ESM.docx]

APPENDIX: Interview Guide

Today, we will talk about your experiences with your need for care, and what you think may have contributed to or been part of why it has sometimes (or often) become very difficult for you, leading you to need emergency or inpatient care.

1. Have you ever reflected on the number of days or visits you have made to the psychiatric emergency department? What have you thought about that?
2. If you try to think of the times over the past year when you have sought help at the psychiatric emergency, or admitted yourself in another way, is there anything that stands out as having happened before that?
   a. Individually: Something that relates to you or your life?
   i. Do you think this is specific to you, or do you think others might also relate to these experiences?
   b. Structurally: Something related to the healthcare system?
   i. Your outpatient care clinic?
   1. If yes, what?
   ii. Other healthcare services?
   1. If yes, what?
3. You have often felt so unwell that you needed to be admitted for psychiatric inpatient care. Are there any specific factors within yourself that you think have contributed to this happening?
   a. If the patient doesn't know: For some, it might be personal traits or difficulties they think have played a role. Do you have anything like that yourself??
   b. Are there any factors within the healthcare system that you think or feel have contributed to this situation for you?
   i. Your outpatient clinic?
   1. Do you have any examples?
   ii. Inpatient care?
   1. Examples?
   iii. Other healthcare services?
   1. Examples?
   c. Do you think these factors are connected? (Individual and structural factors, using the patient's own words)
4. Based on the factors you've mentioned, what type of help do you think is most suitable for you or others who find themselves in a situation where they need frequent inpatient care?
   a. Does it vary in different phases? For example, after an emergency visit, after an admission, after the first admission, etc.
   b. Is this specific to you, or do you think others might also experience this?
5. When someone feels as unwell as you have and ends up in a situation where they need recurrent inpatient care, it often becomes a vicious cycle that is hard to break. What do you think you need or would have needed help with to break that cycle?
   a. Is there something that the healthcare system needs to change?
   i. What? How?
6. How do you think healthcare needs to be adapted or changed to prevent people from ending up in care this frequently and developing such a strong need for emergency and inpatient care?
